# The Nature of Verbal Short-Term Impairment in Dyslexia: The Importance of Serial Order

**DOI:** 10.3389/fpsyg.2016.01522

**Published:** 2016-10-03

**Authors:** Steve Majerus, Nelson Cowan

**Affiliations:** ^1^Psychology and Neuroscience of Cognition Unit, Department of Psychology, University of Liège, LiègeBelgium; ^2^Department of Psychological Sciences, University of Missouri, Columbia, MOUSA

**Keywords:** short-term memory, verbal, serial order, dyslexia, phonological

## Abstract

Verbal short-term memory (STM) impairment is one of the most consistent associated deficits observed in developmental reading disorders such as dyslexia. Few studies have addressed the nature of this STM impairment, especially as regards the ability to temporarily store serial order information. This question is important as studies in typically developing children have shown that serial order STM abilities are predictors of oral and written language development. Associated serial order STM deficits in dyslexia may therefore further increase the learning difficulties in these populations. In this mini review, we show that specific serial order STM impairment is frequently reported in both dyslexic children and adults with a history of dyslexia. Serial order STM impairment appears to occur for the retention of both verbal and visuo-spatial sequence information. Serial order STM impairment is, however, not a characteristic of every individual dyslexic subject and is not specific to dyslexia. Future studies need to determine whether serial order STM impairment is a risk factor which, in association with phonological processing deficits, can lead to dyslexia or whether serial order STM impairment reflects associated deficits causally unrelated to dyslexia.

## Introduction

Dyslexia is characterized by important and persisting difficulties in acquiring accurate and efficient reading abilities despite normal-range intellectual efficiency (Snowling, 2000). Although the precise underlying factors are still a matter of debate, input phonological processing difficulties are most frequently identified as being impaired in dyslexia, in addition to the difficulties in reading acquisition (Ramus et al., 2003, 2013; Serniclaes et al., 2004; Szenkovits and Ramus, 2005). These phonological processing difficulties are considered to prevent efficient mapping of phonemic and graphemic representations, leading to protracted reading development and slowed reading speed in adulthood, and this for languages with either consistent or inconsistent phonology-to-orthography mappings (Ziegler and Goswami, 2005). A further associated factor is verbal short-term memory (STM) impairment. Verbal STM capacity, as measured by digit span or non-word repetition, is typically reduced in children with dyslexia, and this reduction is still present in adults with a history of dyslexia (Brady et al., 1983; Avons and Hanna, 1995; Snowling et al., 1996). This deficit may represent a contributing factor to dyslexia, by reducing the amount of phonological and graphemic information that can be co-activated during the reading process at a given time, and this especially during the recoding reading process, when grapheme–phoneme mappings are not yet automatized (Gathercole and Baddeley, 1993; Martinez Perez et al., 2012b). The purpose of this article is to review the implications for dyslexia of one aspect of STM, the memory for the serial order or serial positions of items, which has received an increasing research interest over the past 5 years. Word reading, especially at the beginning of reading acquisition, is a sequential process involving the extraction of an ordered mental representation of the letters from the printed word, and the construction of a temporary phonological sequences that matches the letter sequence.

## The Problem of Overlap between Verbal STM and Phonological Processing in Dyslexia

The nature of the link between verbal STM impairment and dyslexia is complicated by the fact that mechanisms that allow information to be maintained for a given duration are themselves dependent upon access to long-term memory (LTM) representations. These LTM representations correspond to representations stored in the language system. Several studies showed that the likelihood of an item being recalled in a verbal STM task is determined partly by the nature of underlying linguistic representations: Words lead to higher recall performance than non-words (Hulme et al., 1991). Even for non-words, linguistic knowledge impacts STM in that non-words with a phonotactic structure more frequent in the language lead to higher recall performance than non-words of low phonotactic frequency (Gathercole et al., 1999; Majerus et al., 2004, 2012). Linguistic knowledge thus appears to be an important determinant of verbal STM. If linguistic representations are poorly developed, verbal STM performance will be directly impacted. In the case of dyslexia, this means that verbal STM impairment could be a consequence of the phonological processing impairment which characterizes dyslexia.

## Item-Order Distinction in STM

Not all aspects of verbal STM, however, depend upon access to the language system. Mainly retention of item information appears to be influenced by long-term knowledge: Linguistic variables such as word frequency and semantic similarity determine the number of items recalled in a STM task, but not the number of items recalled in correct serial order (Nairne and Kelley, 2004). At a theoretical level, item information (i.e., the words of a STM list) is considered to be coded by temporarily activating the underlying language representations; serial order information is often considered to rely on distinct processing systems, based on temporal, spatial, or magnitude codes (Henson, 1998; Page and Norris, 1998; Burgess and Hitch, 1999, 2006; Brown et al., 2000; van Dijck and Fias, 2011; van Dijck et al., 2013). Developmental studies have shown that serial order STM abilities predict lexical and reading development independently of item STM abilities, and are the most robust predictor of lexical and reading abilities (Majerus et al., 2006a; Leclercq and Majerus, 2010; Martinez Perez et al., 2012b). Two recent studies showed that serial order STM abilities assessed in children at third year of kindergarten predict their reading decoding abilities 1 and 2 years later (Martinez Perez et al., 2012b; Binamé and Poncelet, 2016). It follows that the distinction between item and serial order STM abilities may be particularly useful for understanding the nature of verbal STM impairment in dyslexia. If verbal STM in dyslexia is simply a consequence of underlying phonological processing difficulties, then performance for item STM should be particularly impaired. If, on the other hand, there are additional, specific STM deficits in dyslexia, serial order STM should also be impaired.

## Item Versus Serial Order STM in Dyslexia

Next, we review the recent studies that have distinguished item and serial order STM abilities in dyslexia. These studies mainly involve adults with a history of dyslexia as there are currently very few studies that specifically investigated serial order STM in children with dyslexia.

### Item versus Serial Order STM in Children with Dyslexia

A first study making an explicit distinction between item and serial order STM was conducted by Martinez Perez et al. (2012a) in children with dyslexia. The authors used tasks designed to maximize temporary retention of either item or serial order information (**Table 1**). The item STM task was a single non-word repetition task probing the retention of phonological item information. Serial order STM was assessed using a serial order reconstruction task for auditory sequences of familiar words. Martinez Perez et al. (2012a) observed both item and serial order STM deficits, with serial order STM deficits and item STM deficits appearing to be independent; the serial order STM deficit was observed relative to both chronological age and reading age matched control groups, whereas the item STM deficit was observed only relative to the chronological age matched control group. These results were partly replicated by Staels and Van den Broeck (2014) in multilingual children with a diagnosis of dyslexia, by showing also both item and serial order STM difficulties, but, contrary to the study by Martinez Perez et al. (2012a), the deficit in the serial order STM task appeared to be dependent upon the deficit in the item STM task.

**Table 1:** Synthetic presentation of studies included in the review, with description of study characteristics, tasks and main outcome.

Study	Age/N	Language	Source of diagnosis	Reading deficit	Item processing task	Serial order processing task	Outcome
**Children**							
Martinez Perez et al. (2012a)	10.30/22	French	Formal	-1.86^b^	*Delayed item repetition task*: Recall of single, monosyllabic non-words after 6-s filled retention delay.	*Serial order reconstruction task*: Auditory sequences of familiar animal names (2–7 stimuli); reconstruction of the order of the animals using cards	Item STM: DYS = RA < CA Order STM: DYS < RA < CA
Staels and Van den Broeck (2014)	10.75/36	Multilingual, Dutch-speaking schools	Formal	-1.81^a^	*Delayed item repetition task* : Recall of single, monosyllabic non-words after 6-s filled retention delay	*Serial order reconstruction task*: Auditory sequences of familiar animal names (2–7 stimuli); reconstruction of the order of the animals using cards	Item STM: DYS < CA Order STM: DYS < CA
Staels and Van den Broeck (2014)	Study 2 10.76/25	Dutch	Formal	-1.49^a^	Not applicable	*Serial order learning task*: Hebb-repetition learning for syllable (verbal condition) and dot sequences (visuo-spatial condition); comparison of reproduction performance for repeating and non-repeating sequences	Hebb Verbal: DYS = CA Hebb Visuo-spatial: DYS = CA
**Adults**							
Bogaerts et al. (2015a)	21.22/25	Dutch	Formal	-1.64^a^	Not applicable	*N-back task* : Continuous sequence of pictures of familiar objects; detection of items identical to those presented two items earlier in the sequence, with serial position confusions manipulated via foils occurring in 3-back positions.	DYS < CA
Bogaerts et al. (2015b)	Study 1 20.60/25 Study 2 21.35/18	Dutch	Formal Formal	-1.64^a^ -3.06^a^	Not applicable	*Serial order learning task*: Hebb-repetition learning for syllable sequences; comparison of reproduction performance for repeating and non-repeating sequences.	Hebb Verbal: DYS < CA
Hachmann et al. (2014)	20.80/21	Dutch	Formal	-1.65^a^	*Word probe recognition task*: 13 pictures depicting familiar objects presented sequentially, followed by an auditory word recognition probe. *Visual probe recognition task*: 13 nonsense drawings presented sequentially, followed by a nonsense drawing recognition probe.	*Digit serial order recognition*: Sequentially presented target and recognition probe lists of nine digits each *Visual serial order recognition*: Target and recognition probe sequences of four nonsense drawings each. Target and probe lists differed by the exchange of two adjacent serial positions	Verbal item STM: DYS = CA Verbal order STM:DYS < CA Visual item STM: DYS = CA Visual order STM: DYS < CA
Laasonen et al. (2012)	43.30/30	Finnish	Self-report	-2.19^b^	Not applicable	*Multi-modal sensory sequence recognition*: Pairs of sequences mixing brief auditory and tactile stimuli (3–15 stimuli); non-matching pairs differed by the order of the stimuli within each sequence	Sequence STM : DYS < CA
Martinez Perez et al. (2013)	24.30/31	French	Formal	-1.80^a^	*Study 1*: *Delayed item repetition task* : Recall of single, monosyllabic non-words after 8-s filled retention delay *Study 2*: *Item and order reconstruction task*: Auditory sequences of familiar animal names (5–9 stimuli); reconstruction of the sequences using cards to be selected from a pool of target and non-target stimuli –> wrongly selected cards = item error score *Study 3: Item recognition task*: Auditory sequences of four non-words, followed by a single non-word recognition probe	*Study 1*: *Serial order reconstruction task*: Auditory sequences of familiar animal names (6–9 stimuli); reconstruction of the order of the animals using cards. *Study 2*: *Item and order reconstruction task* –> wrongly placed cards = order error score Study 3: *Serial order recognition task*: Auditory sequences of four non-words, followed by a two-non-word recognition probe, with non-words in the same order as in the target list or not	Study 1: Item STM: DYS < CA Order STM: DYS < CA Study 2: Item STM: DYS < CA Order STM: DYS < CA Study 3: Item STM: DYS = CA Order STM: DYS < CA
Martinez Perez et al. (2015)	23.60/15	French	Formal	-2.10^b^	*Item recognition task*; *verbal condition*: Array of four horizontally aligned words, followed by a non-word recognition probe; vi*sual condition*: Array of four horizontally aligned unfamiliar faces, followed by a face recognition probe	*Serial order recognition task*; *verbal condition*: Array of four horizontally aligned words, followed by a recognition probe with two non-words aligned vertically; *visual condition*: Array of four horizontally aligned unfamiliar faces, followed by a recognition probe with two faces aligned vertically; participants decided whether the order of the stimuli in the probe display matched their order in the memory array	Verbal item STM: DYS < CA Verbal order STM:DYS < CA Visual item STM: DYS = CA Visual order STM: DYS < CA
Romani et al. (2015)	27.7/44	English	Formal (23) Self-report (13) No diagnosis (8)	-3.42^a^	Not applicable	*Serial order reconstruction task*: Simultaneous or sequential presentation of four Hindi characters or five Japanese characters, followed by a serial order reconstruction task using tiles	Sequential: DYS < CA Simultaneous: DYS = CA
Staels and Van den Broeck (2014)	Study 1 20.69/26	Dutch	Formal	-2.15^a^	Not applicable	*Serial order learning task*: Hebb-repetition learning for syllable (verbal condition) and dot sequences (visuo-spatial condition); comparison of reproduction performance for repeating and non-repeating sequences	Hebb Verbal: DYS = CA Hebb Visuo-spatial: DYS = CA
Szmalec et al. (2011)	21.19/16	Dutch	Formal	-2.65^a^	Not applicable	*Serial order learning task*: Hebb-repetition learning for syllable (verbal condition) and dot sequences (visuo-spatial condition); comparison of reproduction performance for repeating and non-repeating sequences	Hebb Verbal: DYS < CA Hebb Visuo-spatial: DYS < CA
Wang et al. (2016)	Adults/28	English	Self-report	-1.15^a^	*Immediate serial recall task*: recall of Visually presented 6-word lists –> Item score: all items produced on inclusion (‘recall all words’) and exclusion (‘do not recall word in position X’) conditions	*Immediate serial recall task*: –> Order score: all items produced on inclusion divided by Item score	Item STM: DYS = CON Order STM: DYS = CON

*Age/N: age in years and sample size of the DYS group; DYS: group with a formal or self-reported diagnosis of dyslexia; CA: control group matched on chronological age and non-verbal intelligence; RA: control group matched for reading age; CON: control group with no specific matching variable.*

*^a^*Z*-score for performance (speed, combined speed-accuracy, or accuracy) of the dyslexic group on a non-word reading task; ^b^*Z*-score for performance (speed or combined speed-accuracy) of the dyslexic group on a word reading task.*

### Item versus Serial Order STM in Adults with Dyslexia

Studies in adults with a history of dyslexia have used similar study designs to distinguish item and serial order STM abilities. Hachmann et al. (2014) contrasted item and serial order probe recognition tasks, using both verbal and visual STM tasks. They observed specifically impaired serial order STM, and this interestingly for both verbal and visual modalities (**Table 1**). They did not observe verbal item STM impairment, while this impairment could have been expected given that verbal item STM is considered to depend on access to underlying linguistic representations. However, the linguistic levels that are impaired in dyslexia are at the level of phonological rather than lexico-semantic representations (Ramus et al., 2013); the item verbal memory lists in the study by Hachmann et al. (2014) were comprised of sequences of pictures depicting familiar objects followed by an auditory probe word, inducing a strong lexico-semantic component for item maintenance and recognition. Martinez Perez et al. (2013) administered a range of item and serial order STM tasks to adults with a history of dyslexia, and they observed both impaired item STM and serial order STM performances. They did so on the basis of two kinds of results: Those from tasks specifically designed to dissociate item and serial order STM processes, as in Martinez Perez et al. (2012a), and from item and serial order errors observed in an item and serial order reconstruction task (**Table 1**). Item and serial order STM deficits appeared to be statistically independent and their correlation (*r* = -0.07) was non-significant after controlling for verbal and non-verbal intellectual efficiency. Note that the dyslexic participants were impaired in non-word and word item recall tasks, but not in a non-word item probe recognition task, suggesting that phonological item representations were sufficient for accurate recognition but not full reproduction. The finding of specific serial order impairment is also supported by a recent study by Bogaerts et al. (2015a), observing impaired performance in a sample of adult dyslexic participants in an N-back task requiring efficient maintenance and updating of serial order information. In another recent study, however, Wang et al. (2016) found no evidence for either verbal item or serial order STM impairment in undergraduate university students with a self-reported diagnosis of dyslexia. They used a process dissociation procedure to derive item and serial order STM estimates from performance in a word list immediate serial recall task; the task demands of this procedure may have contributed to these results, given that serial order recall is estimated in a rather indirect manner, by asking participants to recall all items except the item occurring in a specified serial position (**Table 1**).

### Brain Correlates of Item and Serial Order STM in Dyslexia

A recent neuroimaging study in adults with a history of dyslexia sheds further light on the status of item and serial order STM in dyslexia. Martinez Perez et al. (2015) investigated the neural networks associated with item and serial order short-term probe recognition tasks for both verbal (words) and visuo-spatial (faces) stimuli (**Table 1**). Although at the behavioral level, item and serial order STM deficits of similar severity were observed for the verbal STM modality, they were associated with distinct neural networks. The dyslexic participants activated to a higher extent the left intraparietal cortex, the bilateral cingulate cortex and the right dorsolateral prefrontal cortex in the verbal item STM condition; this network has been associated with attentional control processes during STM tasks (Silton et al., 2010), and may have reflected the greater difficulty of this task in the dyslexic group relative to the control group. In the serial order STM condition the dyslexic group activated to a lower extent a network centered around the right intraparietal sulcus, which had been identified in other studies to be specifically associated with serial order STM processes (Majerus et al., 2006b; see also Beneventi et al., 2009, for similar results comparing single letter versus letter sequence probe recognition tasks). Interestingly, the same network was also hypoactivated in the visual serial order STM condition, and was, at the behavioral level, associated with both serial order STM and reading impairment. The only condition where the dyslexic participants did not differ from controls, at both behavioral and neural levels, was the visual item STM condition.

### Dyslexia and Serial Order Processing in Other Domains

Other studies have investigated serial order processing capacities in dyslexia using tasks that slightly differ from STM tasks. Szmalec et al. (2011) as well as Bogaerts et al. (2015b) showed that adults with dyslexia present difficulties in learning verbal and visuo-spatial sequences in Hebb repetition experiments involving the reproduction of repeating and novel sequences of supra-span length (**Table 1**). Staels and Van den Broeck (2015) could not replicate sequence learning difficulties in verbal or visuo-spatial Hebb learning experiments, but like Bogaerts et al. (2015b), they had observed impaired performance already for non-repeating, filler sequences, further highlighting the difficulties for temporary maintenance of serial order information in dyslexia. Other studies also point to serial order processing difficulties in dyslexia. Romani et al. (2015) required adult dyslexic participants to reproduce the order of presentation of visual characters (**Table 1**). They observed difficulties in reconstructing the order of the characters, especially when they were presented in a sequential rather than a simultaneous manner. Finally, Laasonen et al. (2012) observed deficits in adults with a history of dyslexia for STM for audio-tactile sequences (**Table 1**) with a strong serial order processing component, and these deficits correlated with performance on verbal STM tasks.

## Discussion

This mini-review of STM deficits in dyslexia reveals a number of important findings. First, all studies reviewed here, except for one, show verbal STM impairment in dyslexia, and these deficits persist until adulthood. Second, the verbal STM deficits cannot be explained only on the basis of underlying phonological processing impairment, given that both item STM, considered to depend most strongly on phonological processing, and serial order STM aspects appear to be impaired; importantly, serial order STM also appears to be impaired in the visuo-spatial STM domain, further ruling out the possibility that serial order STM would only be the consequence of verbal impairment.

At the same time, the level to which item and serial order STM deficits are independent in the verbal domain has been questioned, some studies showing independent verbal item and serial order STM impairment, while others do not. The use of bilingual populations with an emigration background in some studies (Staels and Van den Broeck, 2014), making proper identification of dyslexia difficult, could have been one contributing factor to these inconsistent finding. Dyslexic group specificities may also be related to the absence of both item and serial order STM deficits reported by Wang et al. (2016). In that study, dyslexic participants were undergraduate students at a university with competitive access; dyslexic applicants with important STM difficulties and ensuing learning difficulties (Gathercole and Alloway, 2006) may have difficulties in reaching the academic grades necessary for entry at university, as acknowledged by the authors.

These observations, however, also stress the likely heterogeneity of dyslexia populations as regards the presence and severity of verbal STM impairment, and particularly serial order STM impairment. We further know that serial order STM impairment is not specific to dyslexia as it has also been observed in other developmental learning disorders such as dyscalculia (Attout and Majerus, 2015). We suggest here that poorly developed serial order STM abilities increase the risk of learning difficulties in different cognitive domains and situations (Leclercq and Majerus, 2010; Jaroslawska et al., 2016). If occurring at the same time as phonological processing difficulties, the serial order STM difficulties will put the child in a particularly difficult situation for efficiently learning and performing sequential mappings between orthography and phonology, leading to the phenotype of dyslexia. However, severe phonological processing difficulties may also lead on their own to a phenotype of dyslexia, even if serial order STM is not impaired.

A further question relates to domain-general factors that could explain serial order STM impairment. A number of studies have shown that children with dyslexia show difficulties in processing temporally organized information, at either fast or normal sequential presentation speeds (Laasonen et al., 2001, 2002; Romani et al., 2015). This finding is important as a number of theoretical models of serial order STM propose that serial order information is encoded using time-based codes (Burgess and Hitch, 1999, 2006; Brown et al., 2000); empirical evidence for this assumption has been provided recently (Hartley et al., 2016). Note, however, that time-based models of serial order STM represent only one among many different theoretical accounts of serial order STM (Hurlstone et al., 2014).

Another domain-general factor that needs to be considered is attentional impairment (Cowan, 1988, 2010, 2016). Sequentially presented items and their associations may depend on focus of attention capacity. Cowan et al. (2013) presented lists of three to nine words to adults with the task of determining the most interesting word from each list. A delayed test showed that associations had formed between adjacent list items when the lists were short enough to fit within the hypothesized scope of the focus of attention (Cowan, 2001) but not for longer lists. Attention could thus mediate memory of serial order. Some studies suggest that at least a subset of children with dyslexia present attentional deficits, and especially in the area of visual attention (Valdois et al., 2004). Lobier et al. (2014) observed reduced right superior parietal activation in dyslexic participants relative to controls like Martinez Perez et al. (2015), and this for a task in which they had to categorize multiple verbal or non-verbal stimuli. However, the hypothesis of general attentional impairment does not fit with the dissociation observed between preserved item and impaired serial order visual STM tasks observed by Hachmann et al. (2014) and Martinez Perez et al. (2015). A related question here is whether the impairment observed for visual serial order STM tasks may have been driven by verbal encoding strategies. The fact that right intraparietal cortical areas, involved in non-verbal spatial and attentional processes, supported the serial order STM impairment in the study by Martinez Perez et al. (2015) speaks against this possibility; also, Hachmann et al. (2014) used difficult-to-verbalize nonsense drawings.

The mechanisms linking serial order STM and reading acquisition also need further exploration. Serial order STM may support ordered storage and output of letter-to-sound conversion processes during early reading acquisition and support the matching of letter serial positions within a letter string with those of visual word forms stored in LTM during visual word identification (Davis, 2010; Martinez Perez et al., 2012b). If serial order STM is causally involved in dyslexia, then dyslexic participants should have specific difficulties in these word decoding stages.

Future studies should focus more specifically on children populations with dyslexia, few studies exploring item and serial order STM in dyslexia having focused on children populations. Also, in order to determine the potential causal involvement of serial order STM deficits in dyslexia, longitudinal study designs need to be used, in order to determine to what extent the STM deficits are predictive of the severity of later reading impairment as opposed to simply reflecting an associated deficit. Also, whether the serial order STM impairment precedes dyslexia or whether it arises at a later age, after the diagnosis of dyslexia, remains an open question. Importantly, future studies need to shed more light on the inconsistencies regarding the status of STM functioning in dyslexia observed in some studies. Given that these inconsistencies may be related to some degree of heterogeneity in dyslexic populations, population characteristics should be reported with as much detail as possible and include information about history of diagnosis, tests used to establish the diagnosis, linguistic, and socio-economic environment as well as a comprehensive characterization of both linguistic and non-linguistic cognitive abilities.

## Author Contributions

All authors listed, have made substantial, direct and intellectual contribution to the work, and approved it for publication.

## Conflict of Interest Statement

The authors declare that the research was conducted in the absence of any commercial or financial relationships that could be construed as a potential conflict of interest.
